# A case report of Kuttner tumor mimicking a malignant tumor, leading to overtreatment

**DOI:** 10.1002/ccr3.4120

**Published:** 2021-05-06

**Authors:** Daisuke Baba, Keiichi Sotome, Ichiro Maeda, Kosuke Takabayashi, Kensuke Kiyomizu, Meiho Nakayama, Kaoru Ogawa

**Affiliations:** ^1^ Department of Otorhinolaryngology Kitasato Institute Hospital Tokyo Japan; ^2^ Department of Breast and Endocrine Surgery Kitasato Institute Hospital Tokyo Japan; ^3^ Department of Pathology Kitasato Institute Hospital Tokyo Japan; ^4^ Department of Otorhinolaryngology Japanese Red Cross Asahikawa Hospital Hokkaido Japan; ^5^ Department of Psychiatry Yoshida Hospital Nobeoka Japan; ^6^ Department of Otorhinolaryngology and Good Sleep Centre Nagoya City University Graduate School of Medicine Aichi Japan; ^7^ Department of Otolaryngology Keio University Hospital Tokyo Japan

**Keywords:** differential diagnosis, dynamic MRI, fine‐needle aspiration cytology, Kuttner tumor

## Abstract

Preoperative diagnosis with multimodal approaches might lead to overtreatment. Cautious understanding of cytology and dynamic contrast‐enhanced magnetic resonance imaging is required when a Kuttner tumor is cited as differential diagnosis.

## Graphical abstract

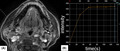

## 1 INTRODUCTION

A case diagnosed as a malignant tumor in the right submandibular gland despite comprehensive workup and performed neck dissection was eventually confirmed a “Kuttner tumor.” To minimize surgical invasion, an opaque finding of submandibular swelling can be a clue indicating Kuttner tumor.

A comprehensive workup with multimodal approaches is the key to a preoperative diagnosis of submandibular gland disease. Fine‐needle aspiration cytology (FNAC) is still one of the most common methods used for preoperative diagnosis.^1^ Meanwhile, dynamic contrast‐enhanced magnetic resonance imaging (DCE‐MRI) is a relatively novel method to differentiate a malignant tumor (MT) from a benign tumor (BT).^2^ An increased variety of diagnostic procedures enables a more accurate and comprehensive preoperative analysis. However, these modalities have some limitations. For example, the heterogeneity and low incidence of salivary gland disease prevent pathologists from gaining sufficient experience. Additionally, reports on differential diagnosis using DCE‐MRI are lacking. Because of these limitations, submandibular gland diseases are sometimes misdiagnosed preoperatively.

A Kuttner tumor (KT) is an easily misdiagnosed type of sialadenitis. To alert all head and neck surgeons, we report a case of KT in which an accurate preoperative diagnosis could not be accomplished despite a comprehensive workup.

## 2 CASE REPORT

An 85‐year‐old woman presented with a palpable mass in her right submandibular triangle, which had appeared 3 months prior. She was on medication for hypertension and rarely complained of tenderness. On clinical examination, the mass was stiff and not fixed either to the surrounding skin or to the floor of the mouth. There were no apparent motor or sensory dysfunctions.

**FIGURE 1:**
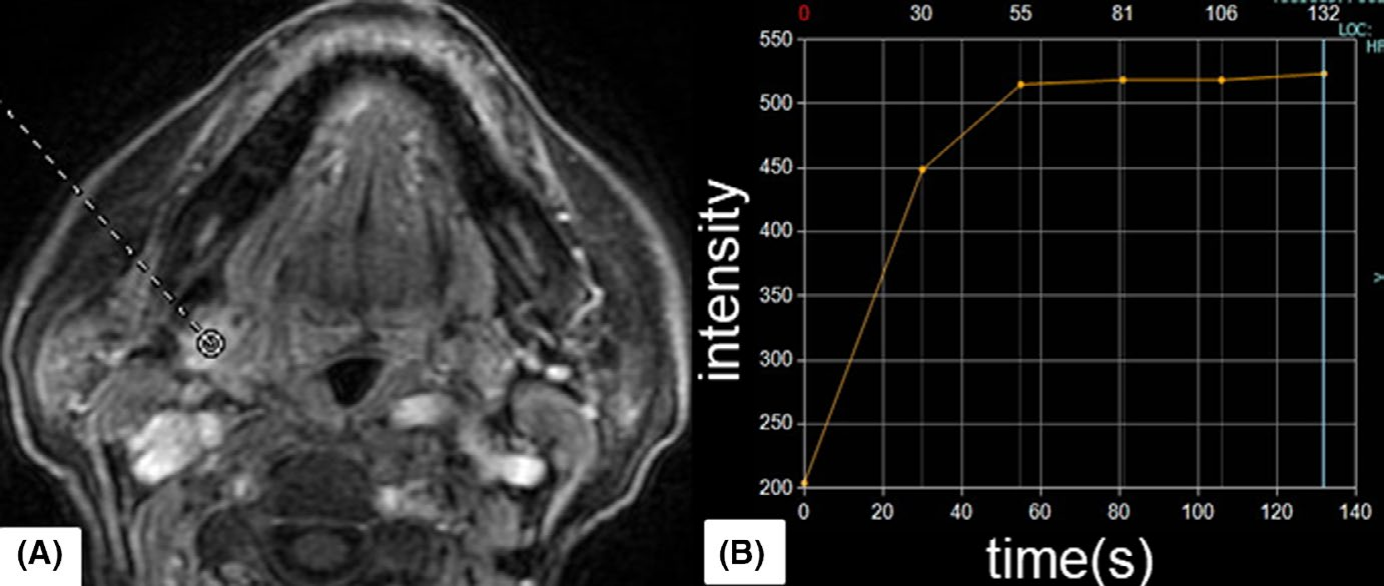
A, Selected area for time‐signal intensity curve (TIC) of dynamic contrast‐enhanced MRI is shown with dotted circle. B, TIC shows a plateau enhancement pattern (type‐C), peak time <120 s, and wash‐out ratio <30%

**FIGURE 2:**
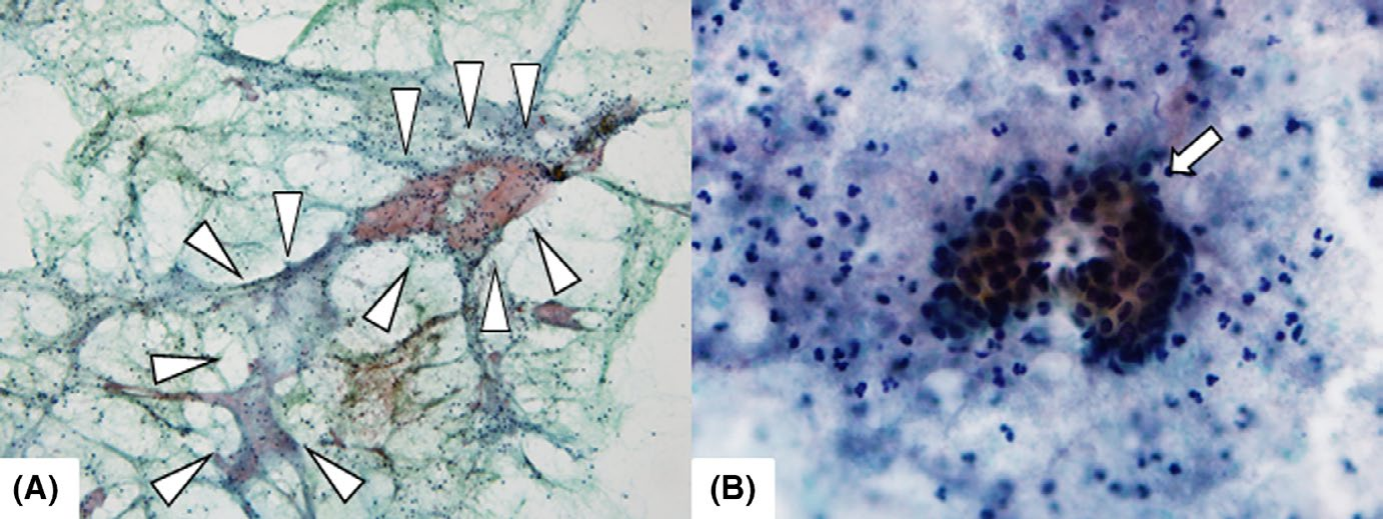
A, Fine‐needle aspiration shows low cellularity of epithelial cells and necrotic‐like substance (arrow head). B, Epithelial cells are surrounded by small lymphocytes and neutrophils and show no nuclear atypia (arrow)

Dynamic contrast‐enhanced magnetic resonance imaging of the right submandibular gland showed a pattern characteristic of an MT (type‐C time‐intensity curve TIC; Figure 1). FNAC showed the presence of a necrotic‐like substance behind lymphoid cells, neutrophils, and a few ductal acini. The cytologic diagnosis indicated that a neoplasm could not be excluded (Figure 2). Based on the results of FNAC and DCE‐MRI, the submandibular mass was diagnosed as an MT.

**FIGURE 3:**
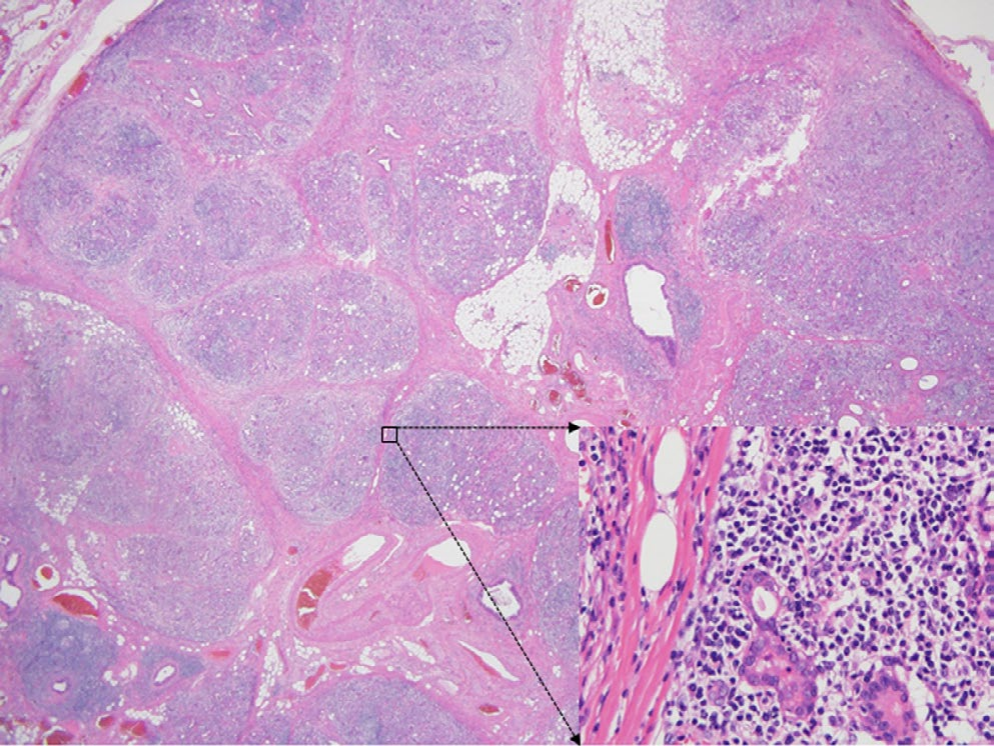
Low‐power magnification shows lobular architecture of salivary gland surrounded by fibrosis (H&E). Selected area of high‐power magnification shows ductal acini structure with lymphocytic infiltration (H&E)

A right neck dissection was performed at level IB. During the procedure, we found that the mass was not adhered to the adjacent muscles and nerves. The gross and histologic findings revealed that the submandibular gland was composed of prominent fibrosis and dense lymphocytic and neutrophilic infiltrates; it also had a ductal acinar structure without atypia. Based on these results, the submandibular lesion was diagnosed as a KT (Figure 3). The postoperative course was uneventful.

## 3 DISCUSSION

We reported a case of overtreatment in which a KT was not accurately diagnosed despite a multimodal preoperative approach, which unfortunately led to neck dissection.

For preoperative diagnosis, FNAC is still the most commonly used method. In a review of FNAC of salivary gland disease, Wei et al reported that the value for MT sensitivity was 87%, while the specificity was 85%.^1^ On the other hand, DCE‐MRI is a relatively novel method first described by Yabuuchi et al^2^ to differentiate MTs from BTs using the TIC. The TIC consists of the curved line of the signal intensity of the selected lesion at designated times after contrast material administration. The TIC is classified into type‐A through D; type‐C indicates MTs. While both FNAC and DCE‐MRI allow for a more accurate preoperative diagnosis, there is still room for error.

Kuttner tumor is a chronic sialadenitis; its clinical features, such as hard swelling, resemble a malignant neoplasm,^3^ whereas 25%‐43% of submandibular gland tumors consist of MTs.^4^ These features sometimes lead to a misdiagnose of KTs as MTs. Therefore, a comprehensive preoperative workup, that includes DCE‐MRI and FNAC, is required for accurate diagnosis.

Regarding FNAC, Leon et al^3^ described the cytological features of KTs as scattered tubular ductal structures often surrounded by fibrosis, a background rich in lymphoid cells, and paucity or the absence of acini. They also reported challenging cases in which it was difficult to differentiate KTs and MTs based on cytology because of the small amount of cellular material and the absence of clear‐cut atypical features.

With respect to DCE‐MRI, there was only one report differentiating KTs, described by Abu et al, in which KTs showed various TIC patterns and they resulted in the difficulty in differentiating KT from MTs.^5^

In the present case, the results of FNAC were mostly consistent with the features of KT; however, we recognized the submandibular gland swelling as a malignant or benign neoplasm rather than a KT because we focused on the necrotic‐like stroma. In order to make a differential diagnosis of “a malignant or benign neoplasm,” DCE‐MRI was used. Since the mass showed a type‐C pattern with this technique, we misdiagnosed the swelling as MT preoperatively. We refrained from using the core‐needle biopsy in order to avoid dissemination. Moreover, we thought that it would be a low‐grade malignant tumor because it is sometimes difficult to distinguish inflammation from a low‐grade malignant tumor by FNAC. This also applies to a frozen section study, which we did not do.

In hindsight, the differential diagnosis of KT could have been emphasized, due to the observed low cellularity and lack of clear‐cut atypia. DCE‐MRI should have been used only in the case where FNAC suggested a neoplasm strongly, since DCE‐MRI can be misleading because of low discriminating capabilities. Then, we would not hesitate to perform a core‐needle biopsy to avoid dissemination. Furthermore, if the results of a core‐needle biopsy could not reach a definite diagnosis, resection of the submandibular gland only could be performed if there was no adhesion to the adjacent tissue. If they showed an MT, then we could have conducted the additional neck dissection of level IB.

Overtreatment of the surgical procedure could have been avoided if we interpreted these results correctly.

## 4 CONCLUSION

We reported a case of overtreatment of KT despite a multimodal approach for preoperative diagnosis. In order to minimize surgical invasion, KT should be cited as a differential diagnosis more emphatically when the cytology shows low cellularity and lack of clear‐cut atypia; besides, DCE‐MRI should be utilized for differential diagnosis only in the situation where FNAC implies a neoplasm profoundly.

## CONFLICT OF INTEREST

The authors declare no conflicts of interest associated with this manuscript.

## ETHICAL APPROVAL

This study was approved by Institutional Review Board of Kitasato Institute Hospital (No. 20068).
